# Equimolar doses of hypertonic agents (saline or mannitol) in the treatment of intracranial hypertension after severe traumatic brain injury: Erratum

**DOI:** 10.1097/MD.0000000000025953

**Published:** 2021-05-21

In the article, “Equimolar doses of hypertonic agents (saline or mannitol) in the treatment of intracranial hypertension after severe traumatic brain injury”,^[1]^ which appears in Volume 99, Issue 38 of *Medicine*, several changes were made in response to “Letter to the Editor: Equimolar doses of hypertonic agents (saline or mannitol) in the treatment of intracranial hypertension after severe traumatic brain injury”.^[2]^ The p value of Effective doses (n) in Table 3 and throughout the article has been updated to 0.207 and the Ineffective doses has been updated to 1.59 in Table 3. There has also been small updates in the Abstract, Results, Discussion and Conclusion to show the slight difference indicated by the updated p value.
